# The effect of ciprofloxacin prophylaxis during haematopoietic cell transplantation on infection episodes, exposure to treatment antimicrobials and antimicrobial resistance: a single-centre retrospective cohort study

**DOI:** 10.1093/jacamr/dlae010

**Published:** 2024-02-01

**Authors:** Ioannis Baltas, Konstantinos Kavallieros, Giannis Konstantinou, Eirini Koutoumanou, Malick M Gibani, Mark Gilchrist, Frances Davies, Jiri Pavlu

**Affiliations:** Department of Infection, Immunity and Inflammation, Institute of Child Health, University College London, London, UK; Department of Haematology, Hammersmith Hospital, Imperial College Healthcare NHS Trust, London, UK; Faculty of Medicine, Imperial College London, London, UK; Faculty of Medicine, Imperial College London, London, UK; Population, Policy & Practice Research and Teaching Department, Great Ormond Street Institute of Child Health, University College London, London, UK; Department of Infectious Disease, Faculty of Medicine, Imperial College London, London, UK; Department of Infectious Disease, Faculty of Medicine, Imperial College London, London, UK; Department of Infectious Disease, Faculty of Medicine, Imperial College London, London, UK; Department of Infectious Disease, Imperial College NHS Healthcare Trust, St Mary's Hospital, London, UK; Department of Haematology, Hammersmith Hospital, Imperial College Healthcare NHS Trust, London, UK; Faculty of Medicine, Imperial College London, London, UK

## Abstract

**Objectives:**

Fluroquinolone prophylaxis during haematopoietic cell transplantation (HCT) remains contentious. We aimed to determine its effectiveness and association with exposure to treatment antimicrobials and antimicrobial resistance.

**Methods:**

All admission episodes for HCT (*N* = 400 , 372 unique patients) in a tertiary centre between January 2020 and December 2022 were studied. Allogeneic HCT (allo-HCT) recipients received prophylaxis with ciprofloxacin during chemotherapy-induced neutropenia, while autologous HCT (auto-HCT) recipients did not.

**Results:**

Allo-HCT was performed for 43.3% (173/400) of patients, auto-HCT for 56.7% (227/400). Allo-HCT was associated with an average of 1.01 fewer infection episodes per 100 admission days (95% CI 0.62–1.40, *P* < 0.001) compared with auto-HCT. In allo-HCT, the total exposure to all antimicrobials was higher [+24.8 days of therapy (DOT)/100 admission days, *P* < 0.001], as was exposure to ciprofloxacin (+40.5 DOT/100 admission days, *P* < 0.001). By contrast, exposure to meropenem (−4.5 DOT/100 admission days, *P* = 0.02), piperacillin/tazobactam (−5.2 DOT/100 admission days, *P* < 0.001), aminoglycosides (−4.5 DOT/100 admission days, *P* < 0.001) and glycopeptides (−6.4 DOT/100 admission days, *P* < 0.001) was reduced. Enterobacteriaceae isolated during allo-HCT were more resistant to ciprofloxacin (65.5%, 19/29 versus 6.1%, 2/33, *P* < 0001), ceftriaxone (65.5%, 19/29 versus 9.1%, 3/33, *P* < 0.001), other antimicrobial classes. Vancomycin-resistant enterococci were more common in allo-HCT recipients (11%, 19/173 versus 0.9%, 2/227, *P* < 0.001). Inpatient mortality during allo- and auto-HCT was 9.8% (17/173) and 0.4% (1/227). respectively (*P* < 0.001).

**Conclusions:**

Ciprofloxacin prophylaxis in allo-HCT was associated with fewer infection episodes and reduced exposure to treatment antimicrobials. Mortality in auto-HCT remained low. A significant burden of antimicrobial resistance was detected in allo-HCT recipients.

## Introduction

Bacterial infections remain the leading cause of morbidity and mortality in adult patients undergoing haematopoietic cell transplantation (HCT).^1^ Risk of infection is particularly high during allogeneic haematopoietic cell transplantation (allo-HCT), compared to autologous haematopoietic cell transplantation (auto-HCT), due to a plethora of factors, including prolonged chemotherapy-induced neutropenia.^2^ Primary antibacterial prophylaxis with fluroquinolones during the chemotherapy-induced neutropenic period has been shown to be effective in reducing mortality, as well as clinically and microbiologically documented infections.^3^ Despite this, there are concerns about the use of broad-spectrum agents as prophylaxis in HCT, due to rising antimicrobial resistance (AMR) rates and the risk of *Clostridioides difficile* infection.^4^ There is particular concern about fluroquinolone resistance, which rises significantly in transplant centres once prophylaxis is introduced.^5^ For this reason, fluroquinolone prophylaxis in HCT remains contentious.^6^

One important aspect of this AMR debate is the effect of antibacterial prophylaxis on the exposure to non-fluroquinolone treatment antimicrobials. In recent Cochrane metanalysis, antimicrobial use (either prophylactic or therapeutic) was not reported as an outcome in any of the included studies.^3^ If fluroquinolone prophylaxis reduces infection episodes, it should also reduce the courses of other treatment antimicrobials. Within the haematology setting, these regimens usually consist of antipseudomonal penicillins, aminoglycosides, carbapenems and glycopeptides, which constitute important targets for control by antimicrobial stewardship (AMS) programmes.^7^ Therefore the overall effect of fluroquinolone prophylaxis on AMS is currently unclear.

In this study, we describe the infections and the burden of AMR in HCT patients in our centre during the coronavirus 2019 (COVID-19) era and assess the effectiveness of fluroquinolone prophylaxis in reducing bacterial infections. Additionally, we aim to investigate the hypothesis that primary antibacterial prophylaxis with fluroquinolones is associated with reduced patient exposure to treatment antimicrobials.

## Methods

### Ethics

All patients provided consent for participation in non-interventional research at the time of HCT. The study was conducted in accordance with the Ethical Principles for Medical Research Involving Human Subjects outlined in the Declaration of Helsinki and was approved by the Institutional Review Board (REC Reference Number 21/LO/0170).

### Setting and study population

This study was performed in Hammersmith Hospital, a 350-bed tertiary referral hospital, part of Imperial College Healthcare NHS Trust. Hammersmith Hospital offers specialist haematology and HCT services (including matched unrelated donor, matched sibling and haploidentical HCT) in West London and is accredited by the Joint Accreditation Committee ISCT-Europe and EBMT (JACIE). All patients admitted for HCT between 1 January 2020 and 31 December 2022 were included in the study and identified through the local transplant registry. No exclusion criteria were applied.

With regards to transplant procedures, all patients were admitted to hospital in dedicated HCT wards, separated from the rest of hospital clinical areas, and nursed in HEPA filtered positive pressure ventilation isolation side rooms. They remained inpatients from the start of conditioning chemotherapy until engraftment (absolute neutrophil count higher than 0.5 × 10^9^/L, sustained >20 × 10^9^/L platelets and haemoglobin >80 g/L, free of transfusion requirements), or resolution of adverse events, whichever was later. The hospital HCT protocols mandate primary antibacterial prophylaxis with ciprofloxacin for patients receiving allo-HCT while neutropenic, whereas auto-HCT recipients do not receive any antibacterial prophylaxis. Antifungal, antiviral and anti-Pneumocystis/anti-Toxoplasma prophylaxis is also offered as per EBMT guidelines.^8^ In short, during the chemotherapy-induced neutropenic period, voriconazole or posaconazole is given to allo-HCT recipients as well as auto-HCT recipients with germ cell tumours, while the remaining auto-HCT recipients receive fluconazole. Aciclovir prophylaxis is given for 5 weeks from the day of HCT for allo-HCT recipients but only during the chemotherapy-induced neutropenic period for auto-HCT recipients. Co-trimoxazole anti-Pneumocystis and anti-Toxoplasma prophylaxis is given to all HCT until the day of HCT and then resumed post engraftment 5 weeks after HCT.

### Management of febrile neutropenia and suspected infection

When signs and symptoms of infection develop, standard operating procedures mandate holding prophylactic antimicrobials and starting treatment with piperacillin/tazobactam and amikacin for all patients for all infectious syndromes. Teicoplanin is also started if a central venous catheter is present. For patients with penicillin allergy or patients remaining febrile after 72 hours, meropenem is substituted for piperacillin-tazobactam. Initial investigations include paired (aerobic and anaerobic) peripheral and central blood cultures, a throat swab for respiratory viruses and a chest X-ray. Additional cultures may be requested depending on the clinical syndrome. Antimicrobials are reviewed at 48 to 72 hours in consultation with a medical microbiologist with the aim to discontinue them early according to ECIL-4 recommendations.^6^ Resolution of chemotherapy-induced neutropenia is not necessary for the discontinuation of antimicrobials. All patients have rectal screens on admission for carbapenem-resistant organisms (CROs) and with nose and groin swabs for MRSA. Screening swabs are then repeated every 7 days. From March 2020, patients were also screened pre-admission with a symptom checklist and a throat swab polymerase chain reaction for SARS-CoV-2. Testing was then repeated on admission and weekly afterwards. At the end of all infection episodes, ciprofloxacin prophylaxis is resumed according to neutrophils counts.

### Definitions, data sources and measurement

Comorbidities were recorded as outlined by the International Severe Acute Respiratory and Emerging Infections Consortium.^9^ Ethnicity was recorded as White, Asian, Black or Other as defined in the 2021 UK Census.^10^ Neutropenia was defined as an absolute neutrophil count of ≤0.5 × 10^9^ cells/L.^8^ Neutropenic and non-neutropenic fever were defined as previously described.^8,11^ CDC definitions for central line-associated bloodstream infection (CLABSI) and other tissue-focused infections were used but mucosal barrier injury-laboratory confirmed bloodstream infections (MBI-LCBIs) were recorded within neutropenic fever.^12,13^

An infection episode was recorded each time a patient showed signs and symptoms of infection and was started on treatment with antimicrobials. It was expressed as infection episodes per 100 admission days to allow comparison of patients with different length of stays. An invasive bacterial infection was recorded when a bacterial pathogen was isolated from a sterile site. Invasive fungal infection (IFI) was recorded when patients met criteria for probable and proven IFI as previously described.^14^ Infection relapse was recorded when the same pathogen was isolated during a subsequent infection episode more than 14 days and less than 60 days after the treatment for a previous episode had been completed. Polymicrobial infections refer to the growth of more than one pathogen in the same culture or in different cultures taken within 48 hours for the same infectious syndrome. Contamination was determined by the growth of a common commensal organism in a single blood culture, which was not isolated again in repeat blood cultures.^15^ Antimicrobial usage was calculated using days of therapy (DOTs) as previously described, and was expressed as DOTs per 100 admission days.^16^ Antimicrobial susceptibility results were reported using EUCAST breakpoints. All data was extracted from electronic medical records.

### Statistical analysis

Statistical analysis was performed using SPSS v.29 (IBM Corp, Armonk, NY, USA). Univariable comparisons were made using the Student's *t*, Mann–Whitney *U* and Kruskal–Wallis tests for continuous variables and the Pearson’s Chi-squared test for categorical variables, as appropriate. Multivariable linear regression was used to identify risk factors associated with the number of infection episodes per 100 admission days and DOTs per 100 admission days. Influential observations were detected using Cook’s distance, standardized Pearson’s residuals and difference in betas.^17^ Model parsimony was assessed using the Bayesian information criterion. Sensitivity analysis was performed for allo-HCT patients not receiving ciprofloxacin prophylaxis and 95% CIs were calculated using 10 000 bootstrap samples. The level of statistical significance was set at 0.05. No power analysis was performed. This study has been reported according to the Strengthening the Reporting of Observational Studies in Epidemiology (STROBE) guidelines.

## Results

### General cohort characteristics

During the study period, there were 400 admissions for HCT (372 unique patients). Allo-HCT was performed for 43.3% (173/400) of patients, while auto-HCT for 56.7% (227/400). Indications for HCT are shown in Supplementary Table 1 (available as Supplementary data at *JAC-AMR* Online) and conditioning regimens in Supplementary Table 2. A summary of participant characteristics is shown in Table 1. Most demographics and comorbidities were comparable between the two groups. Neutropenia was more prolonged in allo-HCT (19 versus 8 days, *P* < 0.001), as was hospital length of stay (36 versus 22 days, *P* < 0.001). A total of 18 patients died during their admission for HCT, 17 of whom received allo-HCT (9.8% versus 0.4%, *P* < 0.001). Infection (66.7%, 12/18) was the leading cause of mortality as recorded on the medical certificate of cause of death, followed by disease progression (11.1%, 2/18), graft versus host disease (11.1%, 2/18), haemorrhage (5.6%, 1/18) and veno-occlusive disease (5.6%, 1/18). Mortality was particularly high in patients who developed IFI (35.7%, 5/14) hospital-acquired pneumonia (HAP) or ventilator-associated pneumonia (VAP), (26.5%, 9/34), as well as patients with infections from VRE (38.1%, 8/21) and *Stenotrophomonas maltophilia* (62.5%, 5/8).

**Table 1.** Participant characteristics

	All participants (*N* = 400)	Allo-HCT (*N* = 173)	Auto-HCT (*N* = 227)	*P*
Demographics				
Age	55 (43–62)	53 (42–60.5)	55 (44–63)	0.08
Sex				
Male	236 (59%)	112 (64.7%)	124 (54.6%)	0.05
Female	164 (41%)	61 (35.3%)	103 (45.4%)	
Ethnicity				
White	262 (65.5%)	114 (65.9%)	148 (65.2%)	0.48
Asian	93 (23.2%)	44 (25.4%)	49 (21.6%)	
Black	22 (5.5%)	7 (4.1%)	15 (6.6%)	
Other	23 (5.8%)	8 (4.6%)	15 (6.6%)	
Body mass index	26.6 (23.5–30.1)	26.3 (23.4–28.9)	27 (23.5–30.6)	0.12
Comorbidities				
Diabetes mellitus	44 (11%)	20 (11.6%)	24 (10.6%)	0.75
HIV	4 (1%)	0 (0%)	4 (1.8%)	0.08
Obesity	105 (26.3%)	35 (20.2%)	70 (30.8%)	0.02
Renal disease	26 (6.5%)	8 (4.6%)	18 (7.9%)	0.18
Renal dialysis	3 (0.8%)	1 (0.6%)	2 (0.9%)	0.73
Respiratory disease	7 (1.8%)	4 (2.3%)	3 (1.3%)	0.45
Asthma	36 (9%)	20 (11.6%)	16 (7%)	0.12
Cardiac disease	50 (12.5%)	16 (9.2%)	34 (15%)	0.09
Liver disease	10 (2.5%)	4 (2.3%)	6 (2.6%)	0.83
Neurological disease	29 (7.2%)	2 (1.2%)	27 (11.9%)	<0.001
Solid neoplasm	7 (1.8%)	5 (2.9%)	2 (0.9%)	0.13
Rheumatological disease	8 (2%)	5 (2.9%)	3 (1.3%)	0.27
Karnofsky score	100 (100–100)	100 (90–100)	100 (90–100)	0.35
Outcomes				
Neutropenia length (days)	11 (7–19)	19 (14–26)	8 (7–10)	<0.001
Length of stay in hospital (days)	27 (21–36)	36 (31–43.5)	22 (19–26)	<0.001
Intensive care admission	32 (8%)	19 (11%)	13 (5.7%)	0.06
Death as inpatient	18 (4.5%)	17 (9.8%)	1 (0.4%)	<0.001

Continuous variables are presented as median (interquartile range), categorical variables as *N* (%).

### Infections during HCT

Only 3.3% (13/400) of patients did not develop an infection episode during their admission (Table 2). Overall, the incidence of infection episodes was lower in allo-HCT (median 4 versus 5 per 100 admission days, *P* < 0.001). An invasive bacterial infection was documented in 31.3% (125/400) of cases. Allo-HCT patients were more likely to have an invasive Gram-positive bacterial infection (40/173, 23.1% versus 29/227, 12.8%, *P* = 0.01), while a difference was not observed for invasive Gram-negative bacterial infections (34/173, 19.7% versus 42/227, 18.5%, *P* = 0.77). *Clostridioides difficile* infection was significantly less common in allo-HCT (1.2% versus 7%, *P* = 0.01). In total, 7% (28/400) of all patients tested positive for CROs during routine screening, 25% (7/28) in their admission screen and 75% (21/28) in hospital after a negative admission screen: 8.5% (34/400) of all patients tested positive for respiratory viruses during their admission, of which two were SARS-CoV-2, both in March 2020 (Supplementary Figure 1).

**Table 2.** Summary of infections and antimicrobials

	All participants (*N* = 400)	Allo-HCT (*N = *173)	Auto-HCT (*N* = 227)	*P*
Types of infection				
Infection episodes per 100 admission days	4.8 (3.6–5.9)	4 (2.9–5.7)	5 (4.2–5.9)	<0.001
Neutropenic fever	279 (69.8%)	118 (68.2%)	161 (70.9%)	0.56
Non-neutropenic fever	96 (24%)	59 (34.1%)	37 (16.3%)	<0.001
HAP or VAP	34 (8.5%)	22 (12.7%)	12 (5.3%)	0.01
CLABSI	57 (14.2%)	32 (18.5%)	25 (11%)	0.04
Intrabdominal infection	32 (8%)	10 (5.8%)	22 (9.7%)	0.15
Urinary tract infection	11 (2.8%)	5 (2.9%)	6 (2.6%)	0.88
Skin and soft tissue infection	10 (2.5%)	6 (3.5%)	4 (1.8%)	0.34
Other infection	2 (0.5%)	0 (0%)	2 (0.9%)	0.22
Invasive bacterial infection	125 (31.3%)	61 (35.3%)	64 (28.2%)	0.13
Invasive bacterial infection—Gram-negative	76 (19%)	34 (19.7%)	42 (18.5%)	0.77
Invasive bacterial infection—Gram-positive	69 (17.3%)	40 (23.1%)	29 (12.8%)	0.01
IFI	14 (3.5%)	12 (6.9%)	2 (0.9%)	0.001
*Clostridioides difficile* infection	18 (4.5%)	2 (1.2%)	16 (7%)	0.01
Positive rectal screen for CRO	28 (7%)	12 (6.9%)	16 (7%)	0.97
Infection relapse	5 (1.3%)	4 (2.3%)	1 (0.4%)	0.1
Antimicrobial exposure				
Total antimicrobial DOT per 100 admission days	148.1 (120.7–185.7)	165.6 (143.7–192.3)	134.8 (108.7–171.4)	<0.001
Meropenem DOT per 100 admission days	21.5 (0–36.4)	18.9 (0–34.4)	23.5 (0–36.8)	0.39
Piperacillin/tazobactam DOT per 100 admission days	22.7 (10.3–33.3)	19.4 (7.4–31.7)	23.8 (13–35)	0.02
Aminoglycoside DOT per 100 admission days	13 (8.3–17.6)	10 (6.9–16.2)	15 (10–17.6)	<0.001
Glycopeptide DOT per 100 admission days	33.3 (22.5–43.5)	30.8 (17.2–41.9)	34.8 (25–44.4)	0.01
Ciprofloxacin DOT per 100 admission days	6.3 (0–40)	44.4 (22.9–68.4)	0 (0–4.3)	<0.001
Phenoxymethylpenicillin DOT per 100 admission days	4.3 (0–13.6)	0 (0–9.9)	8 (0–16.7)	<0.001
Other antimicrobial DOT per 100 admission days	25 (13.6–38.9)	27 (19.1–49.1)	21.1 (9.5–34.3)	<0.001

Continuous variables are presented as median (interquartile range), categorical variables as *N* (%). Other antimicrobials included all antimicrobials with fewer than five DOTs per 100 admission days.

### Antimicrobial exposure during HCT

With regards to antimicrobial exposure, 92.5% (160/173) of allo-HCT patients received ciprofloxacin prophylaxis during the neutropenic period, while none of the auto-HCT patients did. Ciprofloxacin prophylaxis was not administered in 13 cases due to allergy (76.9%, 10/13) or intercurrent antimicrobial treatment throughout the neutropenic period (23.1%, 3/13). Overall, in univariable analysis, allo-HCT patients had higher cumulative exposure to antimicrobials, as well as significantly higher exposure to ciprofloxacin and other antimicrobials (Table 2). On the contrary, exposure to first- and second-line treatment antimicrobials was lower.

### Microbiology of infections during HCT

**Figure 1.**
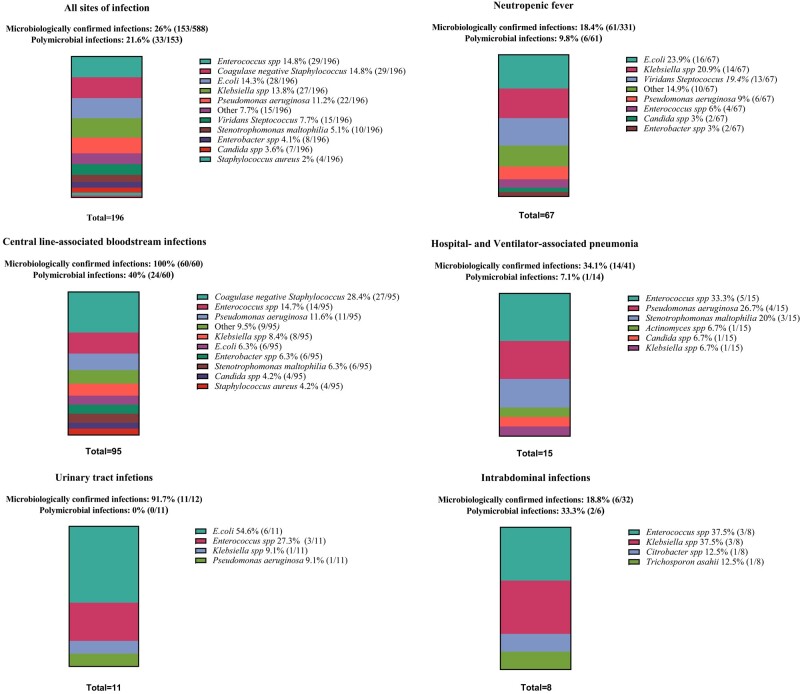
Causes of infection during admission for haemopoietic cell transplantation by site of infection. Strains reported were isolated from blood (82.7%, 162/196), central venous catheter tips (6.1%, 12/196), urine (4.6%, 9/196), bronchoalveolar lavage (3.1%, 6/196), sputum (2.6%, 5/196) and intrabdominal fluid (1%, 2/196). Microbiologically confirmed infections refer to the number of infections with a positive culture. Polymicrobial infections refer to the growth of more than one pathogen in the same culture or in different cultures taken within 48 hours for the same infectious syndrome.

Out of 588 documented infection episodes, 26% (153/588) were microbiologically confirmed (Figure 1). The percentage of microbiologically confirmed infections in allo-HCT versus auto-HCT were similar (26.4%, 80/303 versus 25.6%, 73/285, *P* = 0.99). A total of 196 different pathogens were isolated (82.7%, 162/196 from blood, excluding 24 blood culture isolates that were deemed to represent contamination, rate 8.3 contaminants per 1000 blood cultures). *E. coli* and *Klebsiella* spp. were the leading causes of neutropenic fever, while coagulase-negative *Staphylococcus* was particularly common in CLABSI. *Enterococcus* spp. was frequently implicated in CLABSI and hospital- or ventilator associated pneumonia (HAP or VAP), as was *Pseudomonas aeruginosa.* Only one patient with non-neutropenic fever (1%, 1/100) had a microbiologically confirmed infection. 21.6% (33/153) of all infections were polymicrobial. CLABSIs were a lot more likely to be polymicrobial (40%) than any other site of infection (*P* < 0.001). The rate of CLABSIs was 5.3 infections per 1000 line days.

### Antimicrobial resistance results

**Figure 2.**
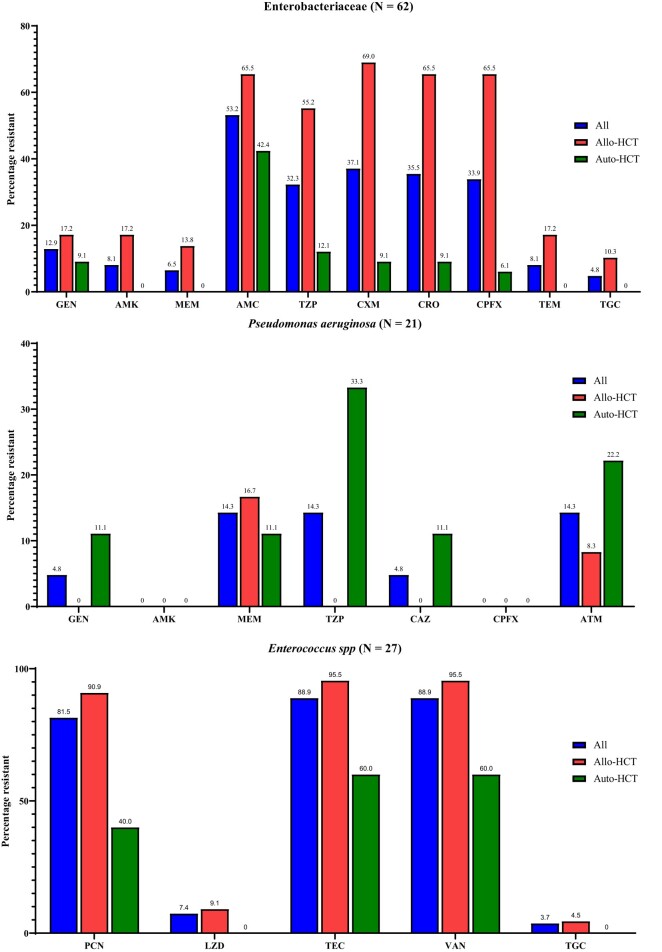
AMR profiles for Enterobacteriaceae, *Pseudomonas aeruginosa* and *Enterococcus* spp. from patients admitted for haemopoietic cell transplantation, reported according to EUCAST breakpoints. Strains reported were isolated from blood (86.4%, 95/110), central venous catheter tips (4.5%, 5/110), bronchoalveolar lavage (4.5%, 5/110), sputum (2.7%, 3/110) and intrabdominal fluid (1.8%, 2/110). GEN, gentamicin; AMK, amikacin; MEM, meropenem; AMC, amoxicillin/clavulanic acid; TZP, piperacillin/tazobactam; CXM, cefuroxime; CRO, ceftriaxone; CPFX, ciprofloxacin; TEM, temocillin; TGC, tigecycline; CAZ, ceftazidime; ATM, aztreonam; PCN, penicillin; LZD, linezolid; TEC, teicoplanin; VAN, vancomycin.

The resistance profiles of Enterobacteriaceae, *Pseudomonas aeruginosa* and *Enterococcus* spp. are shown in Figure 2. There were substantial differences between isolates from allo-HCT and auto-HCT patients, especially with regards to fluroquinolone resistance (65.5% versus 6.1% resistance to ciprofloxacin, *P* < 0.001) in Enterobacteriaceae. On the contrary, all *Pseudomonas aeruginosa* isolates were sensitive to ciprofloxacin and amikacin, while resistance levels to ceftazidime were also low (4.8% across all strains). The majority of *Enterococcus* spp. isolated (88.9%, 24/27) were VRE, but levels of resistance to linezolid (7.4%) and tigecycline (3.7%) remained low (Figure 2). Notably, VRE represented 53.5% (24/43) of all streptococci isolated. All coagulase-negative *Staphylococcus* isolates were sensitive to vancomycin and linezolid, while two cases of teicoplanin resistance were observed (6.9%, 2/29). In total, 20% (2/10) of *Stenotrophomonas maltophilia* isolates were resistant to co-trimoxazole. There were no cases (0/4 isolates) of MRSA.

### Multivariable analysis results for infection episodes and exposure to treatment antimicrobials

**Figure 3.**
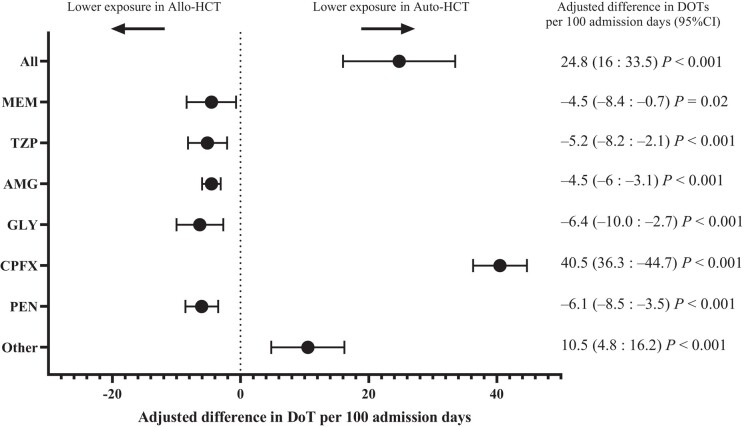
Antimicrobial exposure during admission for HCT. Adjusted multivariable linear regression results for total and individual antimicrobial DOT per 100 admission days between allo-HCT and auto-HCT recipients during admission for HCT. Other antimicrobials included all antimicrobials with fewer than five DOTs per 100 admission days. All results show the estimates of the most parsimonious model. All (antimicrobials): adjusted for age, intensive care admission, obesity, neurological disease, neutropenic fever, non-neutropenic fever, HAP or VAP, CLABSI, intrabdominal infection; MEM (Meropenem): adjusted for neutropenic fever, non-neutropenic fever, HAP or VAP, CLABSI, intrabdominal infection; TZP (piperacillin/tazobactam): adjusted for neutropenic fever, non-neutropenic fever, CLABSI. AMG (aminoglycosides): adjusted for neutropenic fever, non-neutropenic fever, HAP or VAP, CLABSI, intrabdominal infection. GLY (glycopeptides): adjusted for neutropenic fever, non-neutropenic fever, HAP or VAP, CLABSI, intrabdominal infection, neurological disease. CPFX (ciprofloxacin): adjusted for neutropenic fever, non-neutropenic fever, HAP or VAP, CLABSI, intrabdominal infection, neutropenia length. PEN (phenoxymethylpenicillin): unadjusted. Other (antimicrobials): adjusted for HAP or VAP, intrabdominal infection, intensive care admission.

Influential variable analysis identified one allo-HCT recipient who had a short 4-day admission and died of infection during transplantation conditioning chemotherapy. The patient was excluded from multivariable analysis. A linear regression model showed that auto-HCT was associated with 1.01 (95%CI 0.62–1.40, *P* < 0.001) additional infection episodes per 100 days of admission compared to allo-HCT. No other predictors were included in the model because none improved fitness as determined by Bayesian information criterion (Supplementary Table 3). With regards to antimicrobial exposure, linear regression results of the most parsimonious model for total antimicrobial DOT per 100 admission days are shown in Supplementary Table 4, and for individual antimicrobials in Figure 3. Model fitness estimates are presented in Supplementary Table 3. In multivariable analysis, allo-HCT was associated with significantly larger total antimicrobial exposure (24.8 DOTs per 100 admission days, 95%CI 16–33.5, *P* < 0.001). This was primarily driven by higher exposure to ciprofloxacin and other antimicrobials (Figure 3). On the contrary, allo-HCT patients were independently significantly less likely to be exposed to all four first- and second-line antimicrobials for the treatment of suspected infection (Figure 3). The result was preserved in sensitivity analysis excluding allo-HCT patients who did not receive ciprofloxacin prophylaxis (Supplementary Table 5).

## Discussion

Our study results suggest that ciprofloxacin prophylaxis during allo-HCT was associated with reduced incidence of infection episodes and reduced exposure to first- and second-line treatment antimicrobials compared to auto-HCT patients not receiving ciprofloxacin prophylaxis. *Clostridioides difficile* infection was also less common in allo-HCT. Mortality in auto-HCT patients remained low. Significant AMR burden was detected in allo-HCT patients receiving ciprofloxacin prophylaxis.

Our study results indicate that ciprofloxacin prophylaxis remains effective in reducing infections, especially Gram-negative infections, in a setting of high resistance to fluroquinolones. This is important as many studies were conducted during periods when fluroquinolone resistance was particularly low.^3^ Despite allo-HCT recipients being at significantly higher risk for infection compared to auto-HCT recipients, preferential use of ciprofloxacin prophylaxis in allo-HCT only was associated with reversal of this relationship in our cohort.^2^ Additionally, our study highlights the importance of considering the effect of antibacterial prophylaxis on the exposure to treatment antimicrobials, when assessing overall impact on AMR and AMS in future studies. This is an understudied outcome that has been primarily assessed in paediatric cohorts, where a similar reduction in treatment antimicrobials was also noted.^18–20^ Limited existing data on adult patients is also supportive.^21,22^ During HCT, patients are extremely vulnerable to infection, and will inevitably consume antimicrobials, for prophylaxis or for treatment.^23^ Therefore fluroquinolone prophylaxis might be preferable to using wider spectrum antimicrobials for treatment. The AMS benefits from fluroquinolone prophylaxis during HCT might also be compounded by reduced rates of *Clostridioides difficile* infection, which has previously been described.^18,20^ This is thought to be secondary to fluroquinolone lack of anaerobic activity, in contrast to other antimicrobials, including beta-lactams and glycopeptides, but requires further exploration with dedicated studies.^18,20^ It should be noted, however, that any potential benefits from using fluroquinolones must be weighed against the rare risk of potentially long-lasting or irreversible side effects associated with these drugs.^24^

Our study also highlights the burden of AMR in HCT patients, particularly allo-HCT, even in a low-prevalence setting such as England. Of all isolated Enterobacteriaceae, 6.5% were resistant to meropenem, when the national average is less than 1%.^25^ VRE infections were also common, and associated with high mortality in HCT recipients, indicating a significant role for this pathogen, which is frequently not covered by empirical antimicrobial regimens.^26,27^ High rates of resistance to ciprofloxacin were documented, particularly in allo-HCT patients, suggesting a link with fluroquinolone prophylaxis, as resistant strains would be more likely to cause breakthrough infections. Previous studies have indeed shown an increase in resistance rates and need for second-line antimicrobials once fluroquinolone prophylaxis is initiated.^5,28–30^. Although our two cohorts were not directly comparable with regards to baseline risk for AMR (allo-HCT recipients are likely to have had significant higher previous exposure to antimicrobials and healthcare) and the difference in ciprofloxacin resistance rates cannot be solely attributed to the use of fluroquinolone prophylaxis, this further highlights the challenge of balancing risk and benefits for this intervention.

Strengths of our study include investigating a large recent cohort of HCT patients, especially for a single centre, and describing infections and AMR burden during the COVID-19 era. Patients were well-characterized, owing to access to an electronic medical record and there were no missing data. All eligible patients were recruited, minimizing selection bias, and there was no loss to follow up.

Limitations include the retrospective single-centre design, although this ensured all patients were treated according to the same protocols and by the same staff. We cannot exclude residual confounding when comparing antimicrobial use and infection rates in allo-HCT with auto-HCT patients, although the direction of confounding should have minimized rather than exacerbate underlying differences. A small percentage of allo-HCT patients did not receive ciprofloxacin prophylaxis, yet this did not affect results during sensitivity analysis.

In conclusion, ciprofloxacin prophylaxis during allo-HCT was associated with reduced infection episodes and reduced exposure to treatment antimicrobials compared to auto-HCT patients not receiving ciprofloxacin prophylaxis. AMR rates in allo-HCT were significantly higher. Exposure to treatment antimicrobials should be considered when weighing the risks and benefits of fluroquinolone prophylaxis in HCT.

## Supplementary Material

dlae010_Supplementary_Data

## Supplementary data

Supplementary Figure 1 and Supplementary Tables 1–5 are available as Supplementary data at *JAC-AMR* Online.
