# Supplementary material for: The effect of ciprofloxacin prophylaxis during haematopoietic cell transplantation on infection episodes, exposure to treatment antimicrobials and antimicrobial resistance: a single-centre retrospective cohort study
Source: JAC Antimicrob Resist. 2024 Feb 1;6(1):dlae010. doi: 10.1093/jacamr/dlae010 (PMC10833646; doi:10.1093/jacamr/dlae010)
Supplement: dlae010_Supplementary_Data [file dlae010_supplementary_data.docx]

| Supplementary table 1: Indications for haematopoietic cell transplantation in study participants  (N = 400) | |
| --- | --- |
| Allogeneic haematopoietic cell transplantation (N = 173) | Autologous haematopoietic cell transplantation (N =227) |
| Acute myeloid leukaemia 37% (64/173) | Multiple myeloma 57.7% (131/227) |
| Non-Hodgkin’s lymphoma 16.8% (29/173) | Non-Hodgkin’s lymphoma 12.8% (29/227) |
| Myelodysplastic syndrome 10.4% (18/173) | Multiple sclerosis 10.6% (24/227) |
| Myelofibrosis 9.8% (17/173), | Germ cell tumour 10.1% (23/227) |
| Acute lymphoblastic leukaemia 9.2% (16/173), | Hodgkin’s lymphoma 4.8% (11/227) |
| Chronic myeloid leukaemia 5.8% (10/173) | Other 4% (9/227) |
| Hodgkin’s lymphoma 2.9% (5/173) |  |
| Multiple myeloma 2.9% (5/173) |  |
| Other 5.2% (9/173) |  |

| Supplementary table 2: Hematopoietic cell transplantation conditioning regimens in study participants (N = 400) | | | |
| --- | --- | --- | --- |
|  | All (N =400) | Allo-HCT (N = 173) | Auto-HCT (N =227) |
| Cyclophosphamide | 40.3% (161/400) | 57.8% (100/173) | 26.9% (61/227) |
| Mesna | 38.3% (153/400) | 53.2% (92/173) | 26.9% (61/227) |
| Melphalan | 37.5% (150/400) | 8.7% (15/173) | 59.5% (135/227) |
| Busulfan | 31% (124/400) | 67.1% (116/173) | 3.5% (8/227) |
| Fludarabine | 30% (120/400) | 69.4% (120/173) | 0% (0/227) |
| Methotrexate | 23.5% (94/400) | 54.3% (94/173) | 0% (0/227) |
| Etoposide | 18% (72/400) | 6.9% (12/173 | 26.4% (60/227) |
| Alemtuzumab | 16.3% (65/400) | 37.6% (65/173) | 0% (0/227) |
| Thiotepa | 14.5% (58/400) | 31.8% (55/173) | 1.3% (3/227) |
| Lomustine | 13% (52/400) | 6.9% (12/173) | 17.6% (40/227) |
| Cytarabine | 13% (52/400) | 8.7% (15/173) | 16.3% (37/227) |
| Anti-thymocyte globulin | 10.8% (43/400) | 11% (19/173) | 10.6% (24/227) |
| Carboplatin | 5.8% (23/400) | 0% (0/173) | 10.1% (23/227) |
| Paclitaxel | 5.3% (21/400) | 0% (0/173) | 9.3% (21/227) |
| Idarubicin | 0.5% (2/400) | 1.2% (2/173) | 0% (0/227) |
| Rituximab | 0.3% (1/400) | 0.6% (1/173) | 0% (0/173) |
| Total body irradiation | 4.3% (17/400) | 9.8% (17/173) | 0% (0/227) |
| Total lymph node irradiation | 3.3% (13/400) | 7.5% (13/173) | 0% (0/173) |
| Cranial irradiation | 0.3% (1/400) | 0.6% (1/173) | 0% (0/173) |
| Reduced intensity chemotherapy | 25% (100/400) | 57.8% (100/173) | 0% (0/227) |
| Haploidentical graft | 11.8% (47/400) | 27.2% (47/173) | 0% (0/227) |
| Allo-HCT: Allogeneic haematopoietic cell transplantation; Auto-HCT: Autologous haematopoietic cell transplantation | | | |

| Supplementary table 3: Linear regression fitness | | | | | | | | | |
| --- | --- | --- | --- | --- | --- | --- | --- | --- | --- |
| Dependent variable | Infection episodes | Total antimicrobial DOT | MEM DOT | TZP DOT | AMG DOT | GLY  DOT | CPFX DOT | Pen DOT | Other DOT |
| Baseline model BIC | 1704.5 | 4154.7 | 3540.1 | 3364.9 | 2763.7 | 3458.8 | 3490.1 | 3251.1 | 3872.8 |
| **Variable removed BIC** | | | | | | | | | |
| Sex | 1699.3 | 4149.4 | 3534.2 | 3359.2 | 2759.5 | 3453.6 | 3484.7 | 3245.1 | 3866.8 |
| Obesity | 1698.6 | 4154.6 | 3536.6 | 3359.3 | 2757.8 | 3456.4 | 3484.4 | 3245.1 | 3867.2 |
| Neurological disease | 1703.5 | 4157.3 | 3534.4 | 3359.1 | 2761.2 | 3461.2 | 3484.1 | 3247.1 | 3871.3 |
| Non-Neutropenic fever | NA | 4170.0 | 3541.3 | 3371.5 | 2764.9 | 3472.4 | 3492.9 | 3248.6 | 3866.8 |
| HAP/VAP | NA | 4158.3 | 3555.5 | 3359.7 | 2765.9 | 3459.3 | 3503.2 | 3246.0 | 3873.1 |
| CLABSI | NA | 4160.1 | 3542.2 | 3365.1 | 2781.1 | 3475.7 | 3517.9 | 3245.2 | 3871.6 |
| IFI | 1694.1 | 4150.8 | 3535.2 | 3361.6 | 2762.6 | 3453.0 | 3488.9 | 3245.3 | 3867.1 |
| Age | 1694.0 | 4154.8 | 3535.4 | 3359.0 | 2762.1 | 3456.0 | 3484.2 | 3247.3 | 3867.1 |
| Neutropenia length | 1699.6 | 4149.5 | 3534.1 | 3359.2 | 2760.2 | 3455.3 | 3494.0 | 3247.4 | 3866.9 |
| Transplant type | 1719.7 | 4167.5 | 3537.3 | 3365.7 | 2794.2 | 3456.8 | 3716.4 | 3253.6 | 3877.2 |
| Intensive care admission | 1698.7 | 4154.2 | 3537.3 | 3359.0 | 2759.3 | 3457.3 | 3484.2 | 3245.4 | 3874.4 |
| Intrabdominal infection | NA | 4204.3 | 3546.9 | 3359.3 | 2768.0 | 3551.4 | 3511.8 | 3245.1 | 3888.5 |
| Neutropenic fever | NA | 4163.2 | 3545.1 | 3374.2 | 2800.9 | 3488.8 | 3523.0 | 3245.2 | 3867.0 |
| Final model BIC | 1693.6 | 4139.7 | 3507.5 | 3317.5 | 2736.9 | 3440.3 | 3460.1 | 3191.7 | 3837.9 |
| All dependent variables are expressed per 100 admission days. Baseline model includes the following 13 variables: Sex, Obesity, Neurological disease, non-Neutropenic fever, HAP/VAP, CLABSI, IFI, Age, Neutropenia length, Transplant type, Intensive care admission, Intrabdominal infection, Neutropenic fever. Each variable was the sequentially removed to assess its effect on model fitness as determined by the BIC. The analysis with the lowest BIC was selected for the final model. DOT: Days of therapy; MEM: Meropenem; TZP: Piperacillin/tazobactam; AMG: Aminoglycosides; GLY: Glycopeptides; CPFX: Ciprofloxacin; Pen: Phenoxymethylpenicillin; BIC: Bayesian information criterion; HAP: Hospital-acquired pneumonia; VAP: Ventilator-associated pneumonia; CLABSI: central line-associated bloodstream infection; IFI: Invasive fungal infection. | | | | | | | | | |

| Supplementary table 4 | | |
| --- | --- | --- |
| Predictor | Additional DOTs per 100 admission days | p |
| Age (per year younger) | 0.37 (0.07 – 0.68) | 0.02 |
| Allo-HCT | 24.8 (16 – 33.5) | <0.001 |
| Intensive care admission | 19.7 (2.7 – 36.8) | 0.02 |
| Obesity | 10.5 (1.3 – 19.7) | 0.03 |
| Neurological disease | 26.3 (9.5 – 43) | 0.002 |
| Neutropenic fever | 20.1 (10.3 – 29.9) | <0.001 |
| Non-neutropenic fever | 23.7 (13.5 – 34) | <0.001 |
| HAP or VAP | 24.8 (8.7 – 40.9) | 0.003 |
| CLABSI | 21.8 (9.4 – 34.2) | <0.001 |
| Intrabdominal infection | 60 (44.7 – 75.4) | <0.001 |
| Multivariable linear regression results with Total DOTs per 100 admission days as the dependent variable. The results of the most parsimonious model are shown as determined by the Bayesian Information Criterion. DOT: days of therapy; Allo-HCT: allogeneic haematopoietic cell transplantation; HAP: hospital-acquired pneumonia; VAP: ventilator-associated pneumonia; CLABSI: central line-associated bloodstream infection. | | |

| Supplementary table 5: Sensitivity analysis excluding allo-HCT patients who did not receive ciprofloxacin prophylaxis. | | |
| --- | --- | --- |
| Predictor | Additional infection episodes per 100 admission days | p |
| Auto-HCT | 1.2 (0.72 – 1.51) | <0.001 |
|  |  |  |
| Predictor | Additional Total DOTs per 100 admission days | p |
| Age (per year younger) | 0.4 (0.1 – 0.69) | 0.01 |
| Allo-HCT | 26.6 (18 – 35.2) | <0.001 |
| Intensive care admission | 17.2 (0.1 – 34.3) | 0.05 |
| Obesity | 10.1 (1.1 – 19.1) | 0.03 |
| Neurological disease | 25.2 (8.9 – 41.5) | 0.002 |
| Neutropenic fever | 19 (9.3 – 28.7) | <0.001 |
| Non-neutropenic fever | 24.7 (14.6 – 34.8) | <0.001 |
| HAP or VAP | 25.5 (9.4 – 41.6) | 0.002 |
| CLABSI | 20.8 (8.6 – 33) | <0.001 |
| Intrabdominal infection | 61.7 (46.3 – 77.2) | <0.001 |
| Predictor | Additional MEM DOTs per 100 admission days | p |
| Allo-HCT | -4.6 (-0.7 : -8.4) | 0.02 |
| Predictor | Additional TZP DOTs per 100 admission days | p |
| Allo-HCT | -5.9 (-2.8 : -14.2) | <0.001 |
| Predictor | Additional AMG DOTs per 100 admission days | p |
| Allo-HCT | -4.8 (-3.3 : -6.2) | <0.001 |
| Predictor | Additional GLY DOTs per 100 admission days | p |
| Allo-HCT | -6.4 (-2.8 : -10.1) | <0.001 |
| Predictor | Additional CPFX DOTs per 100 admission days | p |
| Allo-HCT | 43 (37.8 – 48.2) | <0.001 |
| Predictor | Additional Pen DOTs per 100 admission days | p |
| Allo-HCT | -6.2 (-3.8 : -8.8) | <0.001 |
| Predictor | Additional Other DOTs per 100 admission days | p |
| Allo-HCT | -10.1 (-4.4 : -15.8) | <0.001 |
| All models are adjusted for the same variables as for the main analysis. Allo-HCT: allogeneic haematopoietic cell transplantation; Auto-HCT: autologous haematopoietic cell transplantation; DOT: days of therapy; HAP: hospital-acquired pneumonia; VAP: ventilator-associated pneumonia; CLABSI: central line-associated bloodstream infection; MEM: Meropenem; TZP: Piperacillin/tazobactam; AMG: Aminoglycosides; GLY: Glycopeptides; CPFX: Ciprofloxacin; Pen: Phenoxymethylpenicillin. | | |

Supplementary Figure 1: Respiratory viruses isolated in throat swabs of symptomatic patients (N=34, 8.5%,34/400) during haematopoietic cell transplantation. SARS-CoV-2: severe acute respiratory syndrome coronavirus 2.
